# The Association between Mineral and Trace Element Concentrations in Hair and the 10-Year Risk of Atherosclerotic Cardiovascular Disease in Healthy Community-Dwelling Elderly Individuals

**DOI:** 10.3390/nu11030637

**Published:** 2019-03-15

**Authors:** Hye-In Choi, Hae-Jin Ko, A-Sol Kim, Hana Moon

**Affiliations:** 1 Department of Family Medicine, Kyungpook National University Hospital, Daegu 41944, Korea; blbr20@naver.com; 2 Department of Family Medicine, School of Medicine, Kyungpook National University, Daegu 41944, Korea; kasgood@hanmail.net; 3 Department of Family Medicine, Kyungpook National University Chilgok Hospital, Daegu 41404, Korea; tnas1103@naver.com

**Keywords:** aged, cardiovascular diseases, minerals, sodium, trace elements

## Abstract

This cross-sectional analysis included 137 Korean subjects aged 60–79 years. All subjects underwent anthropometric measurements and laboratory tests. Scalp hair samples were obtained from each individual, the concentrations of 36 minerals and trace elements were analyzed, and 16 ratios of elements were calculated. ASCVD risk was estimated using pooled cohort ASCVD risk assessment equations for 10-year risk profiles. The 137 subjects were divided into three risk groups: low (<5%, *n* = 28), intermediate (5% to <7.5%, *n* = 21), and high (≥7.5%, *n* = 88) risk groups. After adjusting for obesity (BMI ≥ 25 kg/m^2^) and sex, Na concentration (mg%) in hair was significantly lower in the low-risk (13.91 ± 7.02) than in the intermediate-risk (47.18 ± 8.08) and high-risk (36.76 ± 3.95) groups (*p* for trend = 0.024). The concentration of K (mg%) in hair was also positively associated with the severity of ASCVD risk (10.50 ± 8.37, 23.62 ± 9.63, 33.31 ± 4.71, respectively; *p* for trend = 0.017), but their differences were not statistically significant (*p* = 0.059). By contrast, the levels of Co, U, and Hg, and the Ca/P and Ca/Mg ratios, were negatively correlated with the severity of ASCVD risk (*p* for trend < 0.05). Mean Na concentration in hair was significantly lower in the low-risk than in the other risk groups. By contrast, Co, U, and Hg concentrations showed significant negative associations with risk severity. Further studies are needed to assess whether dietary modification for trace elements could lower the risk of ASCVD.

## 1. Introduction

Over the past few decades, there has been great interest in exploring the precise roles of minerals and trace elements in human health and various diseases. Minerals and trace elements are essential for preventing disease and promoting health through their diverse biological roles in catalytic, structural, and regulatory functional activities throughout the body, with small amounts of these substances interacting with macromolecules, including pro-hormones, pre-secreted granules, and biological membranes. Because most minerals and trace elements are not produced endogenously or are produced in insufficient amounts, they must be ingested, with a balanced and varied diet required to meet bodily needs and prevent deficiencies in these substances [1].

Guidelines of the American College of Cardiology/American Heart Association (ACC/AHA) have defined atherosclerotic cardiovascular disease (ASCVD) as acute coronary syndrome, previous myocardial infarction, stable angina pectoris, previous coronary artery or other reoperation, ischemic or transient ischemic stroke, or atherosclerotic peripheral artery disease [2]. ASCVD is an important public health issue worldwide, being a major cause of morbidity and mortality, and accounting for the largest expenditure in medical budgets [3]. The underlying pathology in patients with ASCVD is atherosclerosis, which develops over many years and is usually advanced by the time symptoms occur, generally in middle age. Delays in clinical diagnosis increase morbidity and mortality risks, and may result in accidental death of the patient. The identification and modification of risk factors as early as possible may better prevent disease and reduce morbidity in individuals who are and are not diagnosed with cardiovascular disease. The identification of new biomarkers may modify traditional cardiovascular risk algorithms, enabling a more accurate diagnosis, sub-classification, risk assessment, and treatment of these [4,5].

Atherosclerosis is characterized not only by cholesterol deposition in arteries, but involves a chronic vascular inflammatory process, which depends on a balance among pro-inflammatory stimuli, anti-inflammatory activities, and antioxidant defense mechanisms. Studies of the associations between minerals and ASCVD have shown that minerals, at appropriate concentrations, have a significant cardio-protective role due to their ability to modulate antioxidants, anti-inflammatory agents, and immune modulatory activities. New evidence suggests that minerals are important mediators of the development and progression of ASCVD, and that the modification of minerals can reduce the risk of ASCVD, suggesting that minerals and trace elements may act directly or indirectly on the cardiovascular system [3,6]. 

The mineral status of individuals is routinely determined by the analysis of biological samples such as blood, urine, hair, and other tissues, but their concentrations in blood and urine are highly dependent on physiological environmental conditions. Also, because their concentration in blood is controlled by homeostatic mechanisms, the concentration of these substances in serum is often not equal to that of the entire organism. Measurements of trace elements in hair have been successful because hair can provide a more accurate record over time than blood or urine of trace elements, including those associated with the accumulation of chemicals in hair samples used for criminal evidence or for evaluating the consumption of drugs of addiction. Hair is easily collected, conveniently stored, and easily processed for the determination of trace element concentrations. Therefore, human hair is increasingly analyzed to monitor quantitative changes in certain elements inside the body and may be useful for assessing environmental exposure, nutritional status, systematic intoxication, and diagnosis of disease [7]. The analysis of mineral concentrations in hair is not medically diagnostic, but provides a method of evaluating the entire body. Studies have assessed the relationships between the mineral analysis of hair and various disorders, including cancer, kidney disease, fibromyalgia, and osteoarthritis [8,9,10]. 

Although the age-standardized mortality rate of South Korean patients with ischemic heart disease (IHD) has decreased, the crude IHD mortality rate has not, suggesting that the population of South Korea is rapidly aging [11]. It has been estimated that 13.1% of the population of South Korea is aged > 65 years, with this percentage expected to increase to 40.1% in 2060. These findings indicate that the burden of IHD disease will likely increase rapidly in the future, as will the need to identify markers associated with early diagnosis and treatment. The present study therefore analyzed the associations between mineral concentrations in hair and the risk of ASCVD in a healthy, community-dwelling population of elderly Korean subjects to determine whether mineral concentrations in hair can be used as an additional tool to assess the risks of ASCVD in this healthy population.

## 2. Materials and Methods

### 2.1. Study Design and Participants

This cross-sectional study, conducted from April to May 2014, initially recruited 168 relatively healthy, community-dwelling individuals aged 60–79 years through a community center in Daegu, South Korea. Subjects were excluded if they (a) had a history of ASVCD, (b) were being treated with any lipid lowering drug, (c) were taking multivitamin/mineral supplements, (d) had dyed or permed their hair within the previous month, (e) returned incomplete questionnaires, or (f) withdrew their consent during the study. Data from a total of 137 subjects were analyzed. All participants provided written informed consent. The research protocol was approved by the local hospital clinical trial board (IRB protocol no. KNUH 2015-04-022).

### 2.2. Biochemical and Anthropometric Measurements

All participants underwent a full physical examination. Baseline anthropometric parameters were measured using a standard protocol. Height and body weight were measured while the subject was wearing light clothing and no shoes. Standing height was measured to the nearest 0.1 cm using a fixed wall-scale, and weight was measured to the nearest 0.1 kg using a body composition analyzer (Inbody 430, Biospace, Seoul, Korea). Body mass index (BMI) was calculated as body weight divided by height squared (kg/m^2^). Waist circumference was measured, using a flexible tape measure, around the horizontal plane just above both iliac crests at the end of normal expiration, as specified by the guidelines of the National Health and Nutrition Examination Survey. Systolic and diastolic blood pressures were measured with an automatic sphygmomanometer after the subject had sat at rest for at least 10 min.

Blood was drawn after an overnight fast of at least 8 h, and serum samples were obtained by centrifugation. Parameters measured included complete blood count; liver function tests, including concentrations of aspartate aminotransferase (AST), alanine aminotransferase (ALT), and gamma-glutamyl transpeptidase (γ-GTP); and concentrations of total cholesterol, high-density lipoprotein (HDL) cholesterol, low density lipoprotein (LDL) cholesterol, fasting plasma glucose, and glycosylated hemoglobin (HbA1c).

The sociodemographic characteristics of participants, including family history, personal history, smoking status, alcohol consumption, and physical activity, were surveyed by questionnaires and direct interviews. Smoking status was classified as current smoker, ex-smoker, and nonsmoker, regardless of duration. Alcohol consumption was classified as non-drinkers, social drinkers (<8 drinks per week or <6 standard drinks on a single occasion), and heavy drinkers (≥8 drinks per week or ≥6 drinks on a single occasion), with a standard drink corresponding to 10 g of alcohol [12]. Regular physical activity was defined as moderate intensity exercise lasting more than 1 h per week.

### 2.3. Hair Tissue Mineral Analysis (HTMA)

Participants were asked not to dye or perm their hair within eight weeks prior to sample acquisition. Each 0.5–1 g hair sample was cut with clean stainless scissors from the posterior vertex region of the scalp, as close to the scalp as possible. The obtained sample was placed directly into a clean envelope and sent to the USA Trace Elements Inc. (TEI; Dallas, TX, USA) through Korea TEI. Mineral and trace element concentrations were measured using a microwave temperature-controlled digestion technique. Briefly, each 250 mg hair sample was washed with a 1:1 mixture of acetone and alcohol, and the solution was wet-ashed with 2.5 mL HNO_3_ solution in a closed tube at room temperature. The samples were dried for about 1 h in a 60–70 °C dry oven and cooled to room temperature. Each sample was diluted with 25 mL Milli Q water and analyzed using a Perkin-Elmer Mass Spectrometer (SciexElan 6100; Perkin-Elmer Corporation, Foster City, CA, USA). Mineral concentrations are expressed as mg% (mg/100 g of hair). The reference ranges, as determined by US TEI, were comprehensively derived from numerous data and widely used in several studies. Hair mineral analysis yielded concentrations of 39 elements and 16 ratios; these elements consisted of 15 nutritional elements (six minerals and nine essential elements), 15 additional elements, and 9 toxic elements. Because three elements, I (Iodine), Pt (Platinum), and Sb (Antimony), were not detected in any of these samples, this study reports results for only 36 elements.

### 2.4. ASCVD Risk Assessment

The 10-year primary risks of ASCVD in participants were estimated using pooled cohort equations (PCEs), as determined using a web-based calculator [http://my.americanheart.org/cvriskcalculator]. The PCEs were described in the 2013 ACC/AHA Guidelines as predicting absolute 10-year risk of ASCVD, including stroke, among patients aged 40–79 years without pre-existing cardiovascular disease. These equations had been developed based on population groups with diverse racial and geographic distributions to improve previous risk assessment models. These equations included age, untreated systolic blood pressure, antihypertensive therapy status, total cholesterol and HDL cholesterol concentrations, current smoking status, and history of diabetes as covariates. The 137 participants were divided into three risk groups, defined as low-risk (risk < 5%; *n* = 28), moderate-risk (5% ≤ risk < 7.5%; *n* = 21), and high-risk (risk ≥ 7.5%; *n* = 88) groups, according to the ACC/AHA guidelines [13,14].

### 2.5. Statistical Analysis

Continuous variables are presented as mean ± standard deviation (SD), and categorical variables as number (%). General characteristics and the results of HTMA among the three groups were compared using Pearson’s chi-square test and analysis of variances (ANOVA), with a Sidak correction as post-hoc analysis. Linear-by-linear association tests for categorical variables and a general linear model for continuous variables were performed to assess whether there was a linear trend between subject characteristics and the risk of ASCVD in the three groups, with concentrations of minerals in hair analyzed separately for each group. Covariance analysis (ANCOVA) was performed after adjusting for obesity (BMI ≥ 25 kg/m^2^) and sex, based on previous studies analyzing the mineral composition of tissues [15,16,17,18,19]. All statistical analyses were performed using IBM SPSS ver. 21 statistical software (SPSS Inc., Chicago, IL, USA), with a *p* value <0.05 considered statistically significant.

## 3. Results

### 3.1. Baseline Characteristics

This study recruited 137 participants, 26 (16.1%) men and 115 (83.9%) women, with a mean age of 69.77 ± 5.97 years and mean predicted 10-year ASCVD risk of 12.15 ± 8.52%. Of these subjects, 14.6% were current or ex-smokers, 69.3% had hypertension, and 21.9% had diabetes. Baseline characteristics of the subjects are shown in Table 1. Subject age was significantly associated with increased ASCVD risk (*p* for trend < 0.001). Subjects with higher ASCVD risk had a significantly higher waist circumference, systolic blood pressure, diastolic blood pressure, and serum concentrations of fasting glucose and HbA1c (*p* for trend < 0.001). Higher ASCVD risk was also associated with a smaller height (*p* for trend = 0.003) and lower HDL cholesterol concentration (*p* for trend = 0.007).

### 3.2. Hair Tissue Mineral Analysis According to the ASCVD Groups

We performed one-way ANOVA and ANCOVA to compare the concentrations and ratios of minerals and trace elements in the three groups stratified according to the severity of predicted 10-year ASCVD risk (Table 2, Table 3, Table 4 and Table 5). The levels of all minerals, except for Ca (Calcium), K (Potassium), Se (Selenium), Rb (Rubidium), Sr (Strontium), Sn (Tin), and Ti (Thallium), were within reference ranges. The mean ± SD levels of Ca, K, Rb, Sr, Sn, and Ti in hair were 127.45 ± 88.67, 27.16 ± 44.78, 0.02 ± 0.03, 1.31 ± 2.42, 0.04 ± 0.06, and 0.17 ± 0.15 mg%, respectively, all exceeding their reference ranges. The concentration of Se in hair was 0.04 ± 0.02 mg%, which was below the reference range. The levels of all toxic elements were within their normal ranges.

After adjusting for obesity (BMI ≥ 25 kg/m^2^) and sex, the mean concentration of Na (Sodium) in hair was significantly lower in the low-risk group than in the intermediate- and high-risk (*p* = 0.004, *p* for trend = 0.024) groups. The concentration of K in hair was positively associated with the severity of ASCVD risk, but the difference among the three groups was not statistically significant (*p* = 0.059, *p* for trend = 0.017). By contrast, the levels in hair of Co (Cobalt) (*p* = 0.011, *p* for trend = 0.008), U (Uranium) (*p* = 0.003, *p* for trend = 0.001), Hg (Mercury) (*p* = 0.025, *p* for trend = 0.046), Ca/P (Phosphate) (*p* = 0.033, *p* for trend = 0.015), and Ca/Mg (Magnesium) (*p* = 0.042, *p* for trend = 0.017) were negatively correlated with the severity of ASCVD risk. The levels of hair Rb (Rubidium) and S (Sulfur)/Hg were positively correlated with risk severity and negatively correlated with Se (Selenium) levels, but the differences among the groups were not statistically significant (*p* ≥ 0.05, *p* for trend < 0.05). Comparisons of all other elements and ratios among the three groups were not statistically significant.

## 4. Discussion

This study utilized PCEs to investigate the relationship between mineral and trace element composition in hair and predicted 10-year ASCVD in subjects aged 60–79 years. The Na concentration in hair samples was lower, whereas the Co, U, and Hg concentrations were significantly higher, in the low-risk group than in the other risk groups. These tendencies were also observed after adjustment for obesity and sex.

Previous epidemiologic and observational studies have shown a positive association between Na intake and risk for ASCVDs, including stroke, due to hypertension [20,21]. The pathogenesis of ASCVDs is thought to be related to a high Na intake, which may contribute to the development of atherosclerosis through the induction and activation of vascular endothelial dysfunction, the rennin–angiotensin–aldosterone system, sympathetic nervous system, left ventricular hypertrophy, heart rate, insulin sensitivity, lipid concentrations, and obesity [22,23,24]. Guidelines have therefore recommended a reduction in Na intake to reduce blood pressure, as well as the risks of cardiovascular disease, stroke, and coronary heart disease [25,26]. Several studies have assessed the relationship between dietary Na intake and mineral content in hair. For example, a comparison of Na concentrations in the hair of residents of Peru and the U.S. showed that Na was significantly lower in the hair of Peruvians, perhaps due to their lower intake of dietary Na or the lower concentrations of Na in their drinking water [27]. These results suggest that Na concentration in hair reflects dietary Na intake, with a higher Na intake associated with an increased risk of ASCVD.

Aldosterone and the vascular mineralocorticoid system are involved in the pathogenesis of vascular inflammation and atherosclerosis, beginning with risk factor-induced endothelial dysfunction and inflammation and proceeding to plaque formation, progression, and ultimately rupture plus thrombosis, the cause of acute ischemia. Aldosterone is negatively regulated by salt (NaCl) status and induces vasodilation in normal physiological situations. Inappropriately elevated plasma aldosterone concentrations in subjects with cardiovascular risk factors result in endothelial dysfunction, leading to resistant hypertension, atherosclerosis, and coronary heart disease [28]. Aldosterone in the kidneys activates apical epithelial sodium channels (ENaCs) and basolateral Na+/K+ ATPase pumps, thereby regulating Na excretion by the distal tubules, and thereby regulating Na homeostasis [29]. ENaC has been identified in epithelial cells of non-transport epithelial tissue, such as human keratinocytes and hair follicles, as well as in various epithelial cells that reabsorb Na, including cells in the collection tubules of the kidneys, the distal colon, and the airways [30]. A large prospective cohort study found that plasma aldosterone concentration is independently associated with ASCVD, and that high-normal to high plasma aldosterone concentrations and high aldosterone rennin ratios contribute to impaired flow-mediated dilation and the subsequent progression of subclinical atherosclerosis [31,32]. These findings suggest that increased aldosterone levels and activity in high-risk ASCVD patients may lead to an increase in intracellular Na uptake via ENaC in hair follicles, resulting in higher Na concentrations in hair. Similarly, Na levels were reported to be higher in the hair of ischemic stroke patients [33] and in blood and scalp hair samples of patients with myocardial infarction [34]. 

Previous cross-sectional studies showed that the Na concentration in hair was higher in subjects with than without metabolic syndrome and associated with insulin resistance [35,36,37]. The prevalence of coronary heart disease was markedly higher in subjects with than without metabolic syndrome [38,39], with the individual components of the metabolic syndrome also associated with an increased risk of coronary heart disease. The combination of insulin resistance and hyperinsulinemia has been shown to lead to chronic hyperglycemia and dyslipidemia. These factors trigger cellular defects, oxidative stress, and inflammatory responses, which lead to atheroma plaque formation, ventricular hypertrophy, and diastolic abnormalities, resulting in a higher risk of ASCVD that includes obesity, diabetes, hypertension, and coronary artery disease [40,41]. 

Co is a trace element known to be essential for the biosynthesis of vitamin B12, which is necessary to produce red blood cells and for maintaining the nervous system [18]. In our study, Co concentrations in hair were higher in the low-risk than in the other groups. Despite significant negative correlations, the differences among the three groups were very small. Also, the extent to which hair Co reflects body stores of this element and the mechanisms and pathogenesis underlying the effects of Co excess and deficiency remain unknown to date. These findings suggest the need for a larger scale investigation of the potential role of Co.

Widespread use of heavy metals in various industrial, agricultural, medical, and technical applications has raised concerns about the potential effects of these elements on human health and the environment. For example, mean concentrations of Pb (Lead), Cd (Cadmium), and Ni (Nickel) in scalp hair were associated with myocardial infarction, and As (Arsenic), Cd, Ni, and Pb concentrations in hair were associated with the number of myocardial infarctions and the degree of myocardial damage [42,43]. By contrast, the levels of Cr (Chromium), Fe (Iron), Al (Aluminum), Cd, and Ni were lower in patients than in healthy individuals [44]. In our study, all toxic elements except U and Hg were present in the reference range, and statistical differences among the groups were not significant. This may have been due to our study population including people living in a single area, with results reflecting local characteristics, such that the differences between individuals were not significant. Because U is ubiquitous in the environment, general populations are exposed to U in soil, air, water, and food. Although U is both an element and a radioactive material, it has been determined that its adverse health effects are primarily a result of its chemical rather than its radiological toxicity. In the bloodstream, U is associated with red cells, and its clearance is relatively rapid. Renal toxicity is a major adverse effect of U, but this metal also has toxic effects on the cardiovascular system, with concentrations differing between healthy individuals and patients with coronary heart disease [45,46]. The discrepancy in results may be due to methodological difference among studies. 

The mean concentrations of Rb and K in hair were higher than their reference ranges, with higher concentrations being positively associated with ASCVD risk, although the differences among the three groups were not statistically significant. The physiological and pathological roles of Rb in the human body are not fully understood [47]. A high dietary intake of K was associated with decreased blood pressure and risk of related cardiovascular diseases, including ischemic stroke. Elevated dietary K intake increases plasma K concentration, thereby inhibiting free radical formation, smooth muscle proliferation, arterial thrombosis, reactive oxygen species formation, and blood pressure [48,49]. By contrast, a case-control study indicated that the level of K in hair was higher in stroke patients than in control subjects, a finding consistent with our results after adjustment [50].

We found that the average concentration of Ca in hair was higher than the reference range. The Ca/P, Ca/K, and Ca/Mg ratios in hair were also greater than their reference ranges due to the high Ca concentration. Ca concentration in hair was found to correlate positively with atherosclerosis [51]. Our finding, that Ca concentration in hair was much higher than the reference range, may have been due to our study being performed only in subjects aged ≥ 60 years. The Ca/Mg ratio in cells has been reported to be higher in subjects with metabolic syndrome, including those with hypertension and insulin resistance, as well as in subjects with left ventricular hypertrophy and arteriosclerosis, than in controls [52]. Studies assessing serum and hair concentrations of Ca, K, and Mg have shown similar discrepancies. However, only small percentages of these elements are present in serum and act on vessel walls. Moreover, these studies did not evaluate subjects’ diet, despite these elements rapidly fluctuating with dietary and other factors. Although these studies recommended the amount of dietary elements needed to avoid deficiencies, they did not evaluate quantitative effects, suggesting the need for further research.

To our knowledge, this is the first study to investigate the relationship between mineral and trace element concentrations in hair in healthy elderly subjects and ASCVD risk, as predicted by PCEs. It should be emphasized that lifestyle habits, such as a heart healthy diet, regular exercise habits, tobacco product avoidance, and healthy weight maintenance, remain important in promoting health and reducing ASCVD risk, both before and after using lipid lowering medications [13]. A 24-h urine collection is considered as the gold standard method to measure sodium intake, but it is inconvenient to collect 24-h urine samples and it does not reflect the day-to-day variability in the intake of sodium [53]. In this regard, our research has shown that HMTA can conveniently and non-invasively assess concentrations of minerals and trace elements, including Na, which may be useful in determining appropriate lifestyle changes that can reduce the risk of ASCVD.

This study had several limitations. First, the sample size was relatively small, and the subjects were predominantly women aged 60–79 years, which may have resulted in selection bias in assessing mineral and trace element concentrations in all elderly individuals or in the general adult population. However, this factor may be a strength of this study, as it minimized any bias associated with age and environment. Second, this study was cross-sectional in design, making it difficult to determine a causal relation between hair mineral composition and ASCVD risk. Future prospective studies are needed to clarify these relationships. Third, this study did not include a survey on diet, which may affect hair mineral concentrations. Fourth, applying the PCEs to Asian populations can overestimate the risk, suggesting that the accuracy of this study may be improved by using validated tools developed to assess cardiovascular risk in Koreans. Fifth, the rate of hair growth varies from person to person, indicating that the same weight of hair may not represent the same time for the accumulation of elements in hair.

## 5. Conclusions

This study investigated the association between the concentrations of minerals and trace elements in hair and the 10-year severity of ASCVD risk in healthy individuals aged ≥ 60 years. The mean Na concentration in hair was significantly lower in the low-risk than in the other risk groups. By contrast, Co, U, and Hg concentrations showed significant negative associations with risk severity. In addition, this study suggests that hair samples, which are obtained easily and noninvasively, may provide useful prediagnostic information on ASCVD risk, thereby helping in risk assessment and improvement. Our findings suggest the need for further studies assessing whether dietary modification for trace elements could lower the risk of ASCVD. 

## Figures and Tables

**Table 1:** Baseline characteristics of the three groups of subjects stratified by the severity of ASCVD risk.

Characteristics	Total (*N* = 137)	10-Year Predicted ASCVD Risk			*p* *	*p* for Trend ^†^
		Low (<5%) (*N* = 28)	Intermediate (5–7.5%) (*N* = 21)	High (≥7.5%) (*N* = 88)		
**Demographic and lifestyle parameters**						
Age (year)	69.77 ± 5.97	61.50 ± 2.65	66.52 ± 2.54 ^a^	73.17 ± 3.97 ^b,c^	<0.001	<0.001
Female	115 (83.9)	22 (78.6)	18 (85.7)	75 (85.2)	0.624 ^‡^	0.450
Current and ex-smoker	20 (14.6)	3 (10.7)	3 (14.3)	14 (15.9)	0.794	0.504
Mild-to-heavy drinker	49 (35.8)	12 (42.9)	9 (42.9)	28 (31.8)	0.434	0.231
Regular exercise	96 (70.1)	19 (67.9)	14 (66.7)	63 (71.6)	0.870	0.653
**Anthropometric and cardiometabolic parameters**						
Height (cm)	156.83 ± 7.73	160.21 ± 1.00	158.07 ± 5.07	155.46 ± 7.10 ^c^	0.012	0.003
Weight (kg)	58.62 ± 8.79	58.86 ± 9.04	59.89 ± 6.12	58.24 ± 9.30	0.738	0.634
Waist circumference (cm)	84.25 ± 8.46	78.57 ± 9.00	84.10 ± 6.88	86.10 ± 7.87 ^c^	<0.001	<0.001
Body mass index (kg/m^2^)	23.80 ± 2.76	22.92 ± 2.86	23.97 ± 2.21	24.04 ± 2.81	0.166	0.080
Systolic Blood pressure (mmHg)	132.14 ± 15.83	119.54 ± 12.69	126.05 ± 14.06	137.60 ± 14.36 ^b,c^	<0.001	<0.001
Diastolic Blood pressure (mmHg)	76.94 ± 9.85	72.54 ± 9.58	74.67 ± 11.68	78.89 ± 8.97 ^a^	0.006	<0.001
Glucose (mg/dL)	116.75 ± 17.87	100.75 ± 11.75	120.77 ± 24.54	120.89 ± 14.65 ^c^	<0.001	<0.001
HbA1c (%)	5.45 ± 0.81	4.75 ± 0.53	5.63 ± 1.13 ^a^	5.63 ± 0.67 ^c^	<0.001	<0.001
Total cholesterol (mg/dL)	192.45 ± 34.05	183.50 ± 26.02	187.67 ± 36.68	196.43 ± 35.30	0.170	0.062
HDL cholesterol (mg/dL)	56.68 ± 15.56	63.57 ± 17.33	57.05 ± 13.70	54.40 ± 14.88 ^c^	0.024	0.007
LDL cholesterol (mg/dL)	112.27 ± 32.32	97.73 ± 18.00	110.61 ± 36.18	114.64 ± 32.68	0.260	0.116
**Biochemical parameters**						
Hemoglobin (mg/dL)	13.23 ± 1.06	13.59 ± 1.25	13.0 ± 1.05	13.17 ± 0.99	0.110	0.127
AST (U/L)	17.77 ± 7.42	20.96 ± 4.32	25.43 ± 8.91 ^a^	24.18 ± 6.00	0.026	0.050
ALT (U/L)	23.72 ± 6.37	16.29 ± 7.03	20.95 ± 10.79	17.48 ± 6.37	0.076	0.821
γ-GTP (U/L)	17.77 ± 7.41	16.82 ± 11.39	27.52 ± 27.48	23.70 ± 18.95	0.130	0.190
Creatinine ((mg/dL)	0.82 ± 0.21	0.77 ± 1.67	0.79 ± 0.19	0.85 ± 0.23	0.182	0.068

Data are presented as mean ± standard deviation or number (%). Abbreviations: ASCVD, atherosclerotic cardiovascular disease; AST, aspartate transaminase; ALT, alanine aminotransferase; γ-GTP, γ-glutamyl transpeptidase; HbA1c, glycosylated hemoglobin; LDL, low density lipoprotein; HDL, high-density lipoprotein. Alcohol intake was divided into three groups: non-drinkers, social drinkers (<8 standard drinks of 10 g alcohol per week or <6 drinks at a single occasion), and heavy drinkers (≥8 drinks per week or ≥6 drinks at a single occasion). * One-way analysis of variance (ANOVA). ^†^ Linear-by-linear association test for trend. ^‡^ Fisher’s exact test. ^a,b,c^ *p* < 0.05 by post-hoc analysis with the Sidak method for comparisons of the a, high- vs. low-risk; b, high- vs. intermediate-risk; and c, intermediate- vs. low-risk groups.

**Table 2:** Concentrations of fifteen nutritional elements in hair in the three groups of subjects stratified by severity of ASCVD risk.

	Reference Range (mg%)	10-Year Predicted ASCVD risk *			*p* *	10-Year Predicted ASCVD Risk ^†^			*p* ^†^	*p* for Trend ^‡^
		Low	Intermediate	High		Low	Intermediate	High		
Ca	22–97	142.11 ± 96.13	127.57 ± 94.00	122.75 ± 85.43	0.606	147.41 ± 15.73	125.84 ± 18.10	121.48 ± 8.84	0.358	0.171
Mg	2–11	8.68 ± 5.88	9.34 ± 7.17	10.07 ± 9.65	0.747	9.08 ± 1.59	9.21 ± 1.83	9.98 ± 0.89	0.853	0.588
Na	4–36	14.18 ± 12.14	47.10 ± 51.21 ^a^	36.69 ± 37.89 ^c^	0.005	13.91 ± 7.02	47.18 ± 8.08 ^a^	36.76 ± 3.95 ^c^	0.004	0.024
K	2–24	11.68 ± 15.65	23.24 ± 28.62	33.02 ± 52.48	0.081	10.50 ± 8.37	23.62 ± 9.63	33.31 ± 4.71	0.059	0.017
Cu	0.9–3.9	2.75 ± 3.34	2.28 ± 1.94	2.38 ± 6.31	0.939	2.72 ± 1.02	2.28 ± 1.17	2.39 ± 0.57	0.950	0.807
Zn	10–21	16.11 ± 5.12	15.62 ± 4.09	14.53 ± 6.00	0.376	16.16 ± 1.06	15.60 ± 1.22	14.52 ± 0.60	0.356	0.154
P	11–20	13.71 ± 2.48	13.67 ± 1.96	14.35 ± 5.67	0.740	13.68 ± 0.91	13.68 ± 1.05	14.36 ± 0.51	0.731	0.461
Fe	0.5–1.6	0.85 ± 0.33	0.93 ± 0.46	1.13 ± 1.95	0.677	0.86 ± 0.30	0.93 ± 0.35	1.13 ± 0.17	0.691	0.398
Mn	0.01–0.13	0.04 ± 0.12	0.04 ± 0.05	0.03 ± 0.05	0.570	0.04 ± 0.01	0.04 ± 0.02	0.03 ± 0.01	0.567	0.322
Cr	0.02–0.08	0.04 ± 0.01	0.05 ± 0.01	0.04 ± 0.01	0.539	0.04 ± 0.00	0.05 ± 0.00	0.04 ± 0.00	0.384	0.337
Se	0.08–0.18	0.04 ± 0.02	0.04 ± 0.01	0.04 ± 0.02	0.109	0.04 ± 0.00	0.04 ± 0.00	0.04 ± 0.00	0.121	0.045
B	0.02–0.91	0.06 ± 0.05	0.10 ± 0.10	0.44 ± 2.95	0.692	0.06 ± 0.45	0.10 ± 0.52	0.44 ± 0.26	0.688	0.408
Co (×10^−3^)	0.001–0.003	2.39 ± 3.13	1.38 ± 0.80	1.42 ± 0.87 ^a^	0.017	2.43 ± 0.30	1.37 ± 0.35	1.41 ± 0.17 ^c^	0.011	0.008
Mo (×10^−3^)	0.001–0.008	5.21 ± 3.24	7.90 ± 14.34	5.23 ± 2.76	0.188	5.44 ± 1.15	7.84 ± 1.32	5.17 ± 0.65	0.195	0.542
S	2.65–5.34	3.96 ± 0.50	4.02 ± 0.22	4.45 ± 3.81	0.702	1.03 ± 0.58	4.00 ± 0.67	4.43 ± 0.33	0.752	0.491

Abbreviations: ASCVD, atherosclerotic cardiovascular disease; Ca, Calcium; Mg, Magnesium; Na, Sodium; K, Potassium; Cu, Copper; Zn, Zinc; P, Phosphate; Fe, Iron; Mn, Manganese; Cr, Chromium; Se, Selenium; B, Baron; Co, Cobalt; Mo, Molybdenum; S, Sulfur. * One-way analysis of variance (ANOVA) and data are presented as mean ± standard deviation. ^†^ Covariance analysis (ANCOVA) adjusted for obesity and sex, and data are presented as mean ± standard error. ^‡^ General linear model using obesity and sex as covariates. ^a,c^ *p* < 0.05 by post-hoc analysis with the Sidak method for comparisons of the a, high- vs. low-risk; and c, intermediate- vs. low-risk groups.

**Table 3:** Concentrations of thirteen additional elements in hair in the three groups of subjects stratified by severity of ASCVD risk.

	Reference Range (mg%)	10-Year Predicted ASCVD Risk *			*p* *	10-Year Predicted ASCVD Risk ^†^			*p* ^†^	*p* for Trend ^‡^
		Low	Intermediate	High		Low	Intermediate	High		
Li (×10^−3^)	0.001–0.006	4.04 ± 5.73	23.24 ± 96.68	2.80 ± 9.40	0.087	3.87 ± 7.28	23.28 ± 8.38	2.84 ± 4.09	0.089	0.530
V (×10^−3^)	0.002–0.014	5.11 ± 4.01	5.62 ± 3.84	5.36 ± 3.81	0.898	5.04 ± 0.73	5.64 ± 0.84	5.38 ± 0.41	0.857	0.755
Ge (×10^−3^)	0.006–0.011	5.46 ± 1.60	8.24 ± 12.06	6.26 ± 8.22	0.481	5.44 ± 1.55	8.24 ± 1.78	6.27 ± 0.87	0.478	0.849
Ba	≤0.26	0.22 ± 0.20	0.25 ± 0.26	0.20 ± 0.18	0.617	0.23 ± 0.04	0.25 ± 0.04	0.20 ± 0.02	0.548	0.392
Bi (×10^−3^)	≤0.039	5.36 ± 9.02	3.96 ± 2.87	7.85 ± 15.95	0.413	5.15 ± 2.56	4.02 ± 2.95	7.90 ± 1.44	0.391	0.254
Rb	≤0.019	0.01 ± 0.02	0.02 ± 0.02	0.03 ± 0.04	0.104	0.01 ± 0.01	0.02 ± 0.01	0.03 ± 0.00	0.077	0.024
Ni	≤0.10	0.05 ± 0.09	0.05 ± 0.04	0.06 ± 0.06	0.679	0.05 ± 0.01	0.05 ± 0.01	0.06 ± 0.01	0.681	0.584
Tl (×10^−3^)	0.00–0.06	0.50 ± 0.00	0.51 ± 0.44	0.50 ± 0.00	0.062	0.50 ± 0.00	0.51 ± 0.00	0.50 ± 0.00	0.063	0.599
Sr	0.03–0.50	1.11 ± 1.18	2.20 ± 4.22	1.18 ± 2.11	0.195	1.20 ± 0.45	2.18 ± 0.52	1.16 ± 0.25	0.204	0.624
Sn	≤0.03	0.03 ± 0.04	0.04 ± 0.04	0.04 ± 0.07	0.721	0.03 ± 0.01	0.04 ± 0.01	0.04 ± 0.01	0.725	0.439
Ti	≤0.06	0.16 ± 0.13	0.13 ± 0.12	0.18 ± 0.16	0.436	0.16 ± 0.03	0.13 ± 0.03	0.18 ± 0.02	0.452	0.442
W (×10^−3^)	≤0.011	1.21 ± 0.63	1.19 ± 0.51	1.14 ± 0.98	0.904	1.25 ± 0.16	1.18 ± 1.19	1.13 ± 0.09	0.851	0.552
Zr (×10^−3^)	≤0.09	10.36 ± 1.89	12.38 ± 8.89	13.98 ± 14.97	0.405	10.51 ± 2.39	12.33 ± 2.75	13.94 ± 1.35	0.445	0.202

Abbreviations: ASCVD, atherosclerotic cardiovascular disease; Ge, Germanium; Ba, Barium; Bi, Bismuth; Rb, Rubidium; Li, Lithium; N, Nickel; Pt, Platinum; Tl, Thallium; V, Vanadium; Sr, Strontium; Sn, Tin; Ti, Titanium; W, Tungsten; Zr, Zirconium. * One-way analysis of variance (ANOVA) and data are presented as mean ± standard deviation. ^†^ Covariance analysis (ANCOVA) adjusted for obesity and sex, and data are presented as mean ± standard error. ^‡^ General linear model using obesity and sex as covariates.

**Table 4:** Concentrations of eight toxic elements in hair in the three groups of subjects stratified by severity of ASCVD risk.

	Reference Range (mg%)	10-Year Predicted ASCVD Risk *			*p* *	10-Year Predicted ASCVD Risk ^†^			*p* ^†^	*p* for Trend ^‡^
		Low	Intermediate	High		Low	Intermediate	High		
U (×10^−3^)	<0.017	15.41 ± 40.96	4.02 ± 9.81	1.37 ± 2.75 ^c^	0.004	15.71 ± 3.60	3.93 ± 4.14	1.29 ± 2.02 ^c^	0.003	0.001
As (×10^−3^)	<0.020	8.14 ± 9.26	15.71 ± 35.60	11.38 ± 18.81	0.456	7.50 ± 3.94	15.92 ± 4.53	11.53 ± 2.21	0.375	0.542
Be (×10^−3^)	<0.001	1.00 ± 0.00	1.00 ± 0.00	1.02 ± 0.21	0.760	1.00 ± 0.03	1.00 ± 0.04	1.02 ± 0.02	0.776	0.483
Hg	<0.180	0.10 ± 0.05	0.11 ± 0.08	0.08 ± 0.052	0.029	0.10 ± 0.01	0.11 ± 0.01	0.08 ± 0.01 ^a^	0.025	0.046
Cd (×10^−3^)	<0.014	2.11 ± 1.85	1.86 ± 1.15	1.78 ± 1.36	0.590	2.12 ± 0.28	1.85 ± 0.32	1.78 ± 1.16	0.561	0.298
Pb	<0.3	0.11 ± 0.04	0.10 ± 0.03	0.11 ± 0.05	0.750	0.11 ± 0.01	0.10 ± 0.01	0.11 ± 0.01	0.784	0.744
Al	<1.8	0.51 ± 0.27	0.44 ± 0.17	0.47 ± 0.26	0.650	0.51 ± 0.05	0.44 ± 0.06	0.47 ± 0.03	0.593	0.513

Abbreviations: ASCVD, atherosclerotic cardiovascular disease; U, Uranium; As, Arsenic; Be, Beryllium; Hg, Mercury; Cd, Cadmium; Pb, Lead; Al, Aluminum. * One-way analysis of variance (ANOVA) and data are presented as mean ± standard deviation. ^†^ Covariance analysis (ANCOVA) adjusted for obesity and sex, and data are presented as mean ± standard error. ^‡^ General linear model using obesity and sex as covariates. ^a,c^ *p* < 0.05 by post-hoc analysis with the Sidak method for comparisons of the a, high- vs. low-risk; and c, intermediate- vs. low-risk groups.

**Table 5:** Ratios of concentrations of minerals and trace elements in hair of the three groups of subjects stratified by severity of ASCVD risk.

	Reference Range	10-Year Predicted ASCVD Risk *			*p* *	10-Year Predicted ASCVD Risk ^†^			*p* ^†^	*p* for Trend ^‡^
		Low	Intermediate	High		Low	Intermediate	High		
Ca/P	1.4–3.6	13.57 ± 16.02	9.41 ± 7.49	8.95 ± 6.45	0.074	13.97 ± 1.70	9.28 ± 1.95	8.85 ± 0.95 ^a^	0.033	0.015
Na/K	1.40–3.40	2.34 ± 2.58	3.85 ± 7.00	2.13 ± 2.00	0.112	2.33 ± 0.64	3.86 ± 0.74	2.14 ± 0.36	0.114	0.471
Ca/K	2.20–6.20	47.95 ± 69.07	21.43 ± 34.27	23.78 ± 49.21	0.085	49.17 ± 9.68	21.02 ± 11.24	23.49 ± 3.49	0.060	0.042
Zn/Cu	4.00–12.00	10.18 ± 6.71	10.08 ± 4.91	14.46 ± 20.07	0.345	10.10 ± 3.16	10.10 ± 3.64	14.48 ± 1.78	0.342	0.172
Na/Mg	2.00–6.00	2.70 ± 3.50	10.26 ± 12.61	8.48 ± 14.02	0.059	2.48 ± 2.35	10.33 ± 2.71	8.54 ± 1.32	0.047	0.058
Ca/Mg	3.00–1.00	17.71 ± 6.50	15.25 ± 5.13	14.84 ± 5.71	0.076	17.93 ± 1.08	15.18 ± 1.24	14.78 ± 0.61	0.042	0.017
Fe/Cu	0.20–1.50	0.48 ± 0.27	0.64 ± 0.56	0.97 ± 1.65	0.203	0.48 ± 0.26	0.64 ± 0.30	0.97 ± 0.15	0.208	0.079
Ca/Pb (×10^−3^)	>0.084	1.34 ± 0.90	1.49 ± 1.54	1.20 ± 0.86	0.447	1.39 ± 0.18	1.48 ± 0.21	1.19 ± 0.10	0.346	0.228
Fe/Pb	>4.4	7.94 ± 3.36	8.76 ± 4.77	11.14 ± 19.46	0.594	8.02 ± 3.01	8.73 ± 3.46	11.12 ± 1.69	0.610	0.331
Fe/Hg	>22	13.50 ± 15.41	13.42 ± 14.74	19.01 ± 33.37	0.549	13.71 ± 5.34	13.34 ± 6.15	18.96 ± 3.00	0.562	0.987
Se/Hg	>0.8	0.54 ± 0.38	0.56 ± 0.41	0.60 ± 0.38	0.744	0.54 ± 0.07	0.56 ± 0.09	0.60 ± 0.04	0.739	0.444
Zn/Cd (×10^−3^)	>0.500	11.03 ± 5.51	11.52 ± 6.88	11.02 ± 5.71	0.937	10.97 ± 1.12	11.54 ± 1.29	11.03 ± 0.63	0.932	0.965
Zn/Hg (×10^−3^)	>0.200	0.23 ± 0.18	0.20 ± 0.14	0.24 ± 0.12	0.558	0.24 ± 0.03	0.20 ± 0.03	0.24 ± 0.015	0.529	0.743
S/Hg (×10^−3^)	>28.450	53.92 ± 38.92	51.65 ± 35.80	67.69 ± 31.98	0.054	54.33 ± 6.35	51.50 ± 7.30	67.60 ± 3.60	0.054	0.034
S/Cd (×10^−6^)	>0.071	2.85 ± 1.31	2.76 ± 1.38	3.05 ± 1.27	0.570	2.83 ± 0.25	2.77 ± 0.28	3.06 ± 0.14	0.547	0.344
S/Pb (×10^−3^)	>5.690	38.08 ± 8.14	38.12 ± 7.83	39.56 ± 5.10	0.425	10.97 ± 1.12	11.54 ± 1.29	11.03 ± 0.63	0.932	0.274

Abbreviations: ASCVD, atherosclerotic cardiovascular disease; Ca, Calcium; Mg, Magnesium; Na, Sodium; K, Potassium; Cu, Copper; Zn, Zinc; P, Phosphate; Fe, Iron; Mn, Manganese; Cr, Chromium; Se, Selenium; B, Baron; Co, Cobalt; Mo, Molybdenum; S, Sulfur; Pb, Lead; Hg, Mercury; Cd, Cadmium. * One-way analysis of variance (ANOVA) and data are presented as mean ± standard deviation. ^†^ Covariance analysis (ANCOVA) adjusted for obesity and sex, and data are presented as mean ± standard error. ^‡^ General linear model using obesity and sex as covariates. ^a^ *p* < 0.05 by post-hoc analysis with the Sidak method for comparisons of high- vs. low-risk groups.
